# Chronic disease risk factors, healthy days and medical claims in South African employees presenting for health risk screening

**DOI:** 10.1186/1471-2458-8-228

**Published:** 2008-07-04

**Authors:** Tracy L Kolbe-Alexander, Chris Buckmaster, Craig Nossel, Liezel Dreyer, Fiona Bull, Timothy D Noakes, Estelle V Lambert

**Affiliations:** 1 UCT/MRC Research Unit for Exercise Science and Sports Medicine, Department of Human Biology, UCT School of Health Sciences, University of Cape Town, Cape Town, South Africa; 2 Discovery Health Medical Scheme, Johannesburg, South Africa; 3 BHF National Centre for Physical Activity and Health and School of Sports and Exercise Science, University of Loughborough, Loughborough, UK

## Abstract

**Background:**

Non-communicable diseases (NCD) accounts for more than a third (37%) of all deaths in South Africa. However, this burden of disease can be reduced by addressing risk factors. The aim of this study was to determine the health and risk profile of South African employees presenting for health risk assessments and to measure their readiness to change and improve lifestyle behaviour.

**Methods:**

Employees (n = 1954) from 18 companies were invited to take part in a wellness day, which included a health-risk assessment. Self-reported health behaviour and health status was recorded. Clinical measures included cholesterol finger-prick test, blood pressure and Body Mass Index (BMI). Health-related age was calculated using an algorithm incorporating the relative risk for all case mortality associated with smoking, physical activity, fruit and vegetable intake, BMI and cholesterol. Medical claims data were obtained from the health insurer.

**Results:**

The mean percentage of participation was 26% (n = 1954) and ranged from 4% in transport to 81% in the consulting sector. Health-related age (38.5 ± 12.9 years) was significantly higher than chronological age (34.9 ± 10.3 yrs) (p < 0.001). Both chronological and risk-related age were significantly different between the sectors (P < 0.001), with the manufacturing sector being the oldest and finance having the youngest employees. Health-related age was significantly associated with number of days adversely affected by mental and physical health, days away from work and total annual medical costs (p < 0.001). Employees had higher rates of overweight, smoking among men, and physical inactivity (total sample) when compared the general SA population. Increased health-related expenditure was associated with increased number of risk factors, absenteeism and reduced physical activity.

**Conclusion:**

SA employees' health and lifestyle habits are placing them at increased risk for NCD's, suggesting that they may develop NCD's earlier than expected. Inter-sectoral differences for health-related age might provide insight into those companies which have the greatest need for interventions, and may also assist in predicting future medical expenditure. This study underscores the importance of determining the health and risk status of employees which could assist in identifying the appropriate interventions to reduce the risk of NCD's among employees.

## Background

It has been well established that the number of deaths attributable to non-communicable diseases such as coronary artery disease, diabetes and hypertension are increasing globally [1,2]. This trend is also evident in South Africa, where 37% of all deaths are due to non-communicable diseases [2]. However, the burden of non-communicable diseases may be prevented, in part, by addressing certain lifestyle risk factors, including healthy nutrition, regular physical activity and refraining from smoking are all associated with reduced risk for these diseases [3]. The South African Demographic Health Survey (SADHS), conducted in 2002 among the general South African population, showed that 55% and 29% of South African women and men respectively, were overweight [2]. In addition, nearly half of the men and women were insufficiently active and 42% of men and 11% of women were smokers [2].

The workplace has been identified as a likely setting in which to reach a large section of the adult population and positively impact on the health risk profile of individuals [4]. Moreover, the focus of occupational health has shifted in recent years from occupational exposures to non-communicable diseases, and the consequent impact on individual health, and economic costs to companies [5,3]. Kuriyama and colleagues found that physical inactivity, smoking and obesity were associated with an 8.0%, 8.35% and 7.1% increase in health care charges, respectively. In addition, employees having a combination of all three modifiable risk factors had the highest percentage increase (42.6%) in health care expenditure compared to their lower risk counterparts [6]. Further, with an increase in disease burden and absenteeism, there are indirect costs to corporations, which are largely unmeasured [7] and often overlooked.

However, Aldana et al., and others, have demonstrated positive preventative effects of worksite health promotion programs on chronic diseases, [8] with lower rates of absenteeism [8]. Despite this, participation in worksite interventions is generally low [9], and employees who complete worksite interventions are generally healthier than those who drop out [10]. It has also been argued that individuals consenting to medical examinations are more likely to have positive health behaviours than non-participants [11]. Conversely, there were no significant differences in average number of risk factors at baseline between participants and non-participants in Johnson and Johnson's Health and Wellness programme [12]. Lynch et al, also found that responders to health risk assessments were more likely to be younger, receive a salary (versus wage or casual worker) and to file more health-related claims [13].

Despite, the potential for bias with healthier employees more likely to present for health risk assessments, the findings may enable companies to determine the health profile of some of their employees, and also to project future health-care costs. Once risk profiles and employee health problems have been identified, targeted and relevant intervention strategies can be designed and implemented. This may result in subsequent reductions in absenteeism and improve productivity and therefore bring about a reduction in the associated costs [14].

South Africa's largest private health insurer has a programme for their corporate clients which includes wellness days providing employees with an opportunity to have clinical measures such as cholesterol and blood pressure and also to complete a health risk assessment. The present study was undertaken on this existing programme to evaluate and characterise health risk behaviour of employed persons voluntarily presenting for health risk appraisal, as part of employee wellness days conducted by a major national health insurer. The aim of this study was to determine the occurrence of risk factors among employees in the corporate sector, using self-reported health behaviour and clinical measures, of an existing wellness programme, and thereby to inform the development of appropriate and cost-effective intervention strategies.

## Methods

### Participants and sectors

South Africa's largest health insurer offers their corporate clients an opportunity to host wellness days for their employees once per year. This research study was therefore an evaluation of an existing programme and the participants were employees (n = 1954) from eighteen companies where wellness days took place between January and June 2006. All employees from these companies were invited to participate in a one-day health and wellness event. Employees participated on a voluntary basis, and all information gathered remained confidential and was not made available to management or human resource departments.

The wellness days were conducted during normal work hours therefore employees were able to use their normal working time to participate. There were no exclusion criteria, with the only prerequisite for participation being that the individual was an employee of the respective company. Each employee's results were recorded using an unique identity code, and therefore no individual informed consent was signed by each employee. Employees were however aware that the results of the wellness days would be used for research purposes.

Because a unique identity code was allocated by the health insurance administrators, the investigators were not aware of the employee's identity. The health insurance company forwarded the unlinked data results of the health risk assessment and clinical measures to the researchers for data analysis. Ethical approval for this research study was obtained from the Research and Ethics Committee of the Faculty of Health Sciences, from the University of Cape Town.

The companies were conveniently grouped into one of eight business sectors, based on their core profit-generating activities. These sectors included; Engineering (ENG), Logistics (LOG), Consulting (CONS), Information Technology (ITS), Manufacturing (MAN), Academic (ACA), Financial (FIN) and Transport (TRANS).

### Recruitment Procedure and Health Risk Assessment (HRA)

The health and wellness events were advertised one week in advance, and then three, two and 1 days prior to the event using e-mails and the strategic placement of posters within companies. Advertisements emphasised that participation was voluntary and that results would remain confidential. With the exception of one company, all wellness events took place on one day. In a single company, the wellness event occurred over a two and one-half day period.

Participants completed a demographic and lifestyle questionnaire, which assessed smoking status, habitual physical activity, nutrition and self-reported health status and quality of life. Additionally, the health screening was comprised of the finger-prick capillary blood samples for serum cholesterol concentrations, blood pressure, height and weight measurements. All screening was conducted by qualified, trained staff, provided by the health insurer as part of their existing programme.

### Measures

#### Discovery Index Questionnaire

The Discovery Index Questionnaire is comprised of demographic, health and lifestyle factors, as well as questions related to stages of change for the various risk behaviours. The demographic variables include age and gender; while the health and lifestyle measures, include smoking status, fruit and vegetable intake, habitual alcohol consumption and weekly physical activity habits, serum cholesterol, blood pressure, height and weight. Habitual physical activity questions targeted frequency, relative intensity and minimum and maximum duration per session, resulting in an estimated minimum or maximum minutes of moderate-to-vigorous activity and MET (metabolic equivalents) per week.

For smoking, fruit and vegetable intake, alcohol consumption, weight and habitual physical activity, the participant also reported on their readiness to change or improve their lifestyle. The questions on willingness to change were based on the Transtheoretical Model stages of change [15]. The Healthy Days Questionnaire, devised and tested by the US Centers for Disease control, were used to measure health-related quality of life. Healthy days are calculated using a series of 4 questions, focusing on general perceived health, self-rated physical and mental health and the extent to which physical and/or mental health may have limited activity within the past 30 days [16].

#### Risk-related age

The relationship between all cause mortality and elevated cholesterol, BMI, habitual weekly physical activity, fruit and vegetable intake and smoking status, was used to calculate risk-related age. The first step towards developing risk related age comprised of conducting a pub-med search for cohort studies published between 1990 and 2000, which investigated the relationship between the specific risk factor and all cause mortality after adjusting for age, years of education, socio-economic status and co morbidities. A separate search was conducted for each of the following risk factors; smoking, physical activity, fruit and vegetable intake, serum cholesterol concentration and Body Mass Index (BMI). The second step involved the calculation of a pooled relative risk for each risk factor and all cause mortality. The pooled relative risks were then entered into a mathematical model which developed to calculate risk-related age.

The following assumptions were taken into consideration when developing the algorithm to calculate risk-related age; the risk of dying form chronic disease at 20 years of age is 0%; the individual relative risks are independent; the model is only valid for those younger than 70 years; the model is not valid for those who already have a pre-existing condition such as hypertension or diabetes, or those who have already had an "event", such as a myocardial infarction.

Elevated cholesterol, being overweight or obese and smoking could lead to a 'loss' in years and higher risk-related age. Conversely, eating more than five servings of fruit and vegetables per day, and participating in more than 150 minutes of physical activity per week leads to a 'gain' in years, and lowers risk-related age.

#### Classification of Risk Factors

Participants were classified as 'at risk' for the each of the risk factors as follows;

Less than 5 servings of fruit and vegetables per day [10];

Men older than 45 years of age and women older than 55 years;

BMI greater than 25 kg/m^2 ^(overweight) and greater than 30 kg/m^2 ^(obese) [17];

Blood pressure greater than 140/90 mmHg [18];

Cholesterol levels greater than 5.2 mmol/l [12];

Less than 150 minutes/week of moderate-vigorous intensity physical activity [19]

Reporting tobacco use/smoking [12].

### Clinical measures

#### Cholesterol Screening

A finger-prick test (Accutrend ^® ^GC analysers, Roche Diagnostics) was used to measure total serum cholesterol concentrations.

#### Blood Pressure

Blood pressure was measured using an automated sphygmomanometer. Employees were seated for approximately three minutes before being measured.

#### Height and Weight

Standing height (cm) was measured to the nearest 0.1 cm, using a stadiometer. Body mass was measured using a portable calibrated scale and recorded to the nearest 0.5 kg. Body mass index (BMI) was calculated as body mass (kg) divided by height (m) squared (kg/m^2^).

### Statistical Analysis

STATISTICA software package was used for all the analyses (Stasoft, Inc. 184–199, Tulsa OK, USA). Descriptive statistics were performed for the total sample, and separated by business sector and gender. Mean, standard deviation and standard error were calculated for all continuous variables. Because physical activity data were not normally distributed, the median and quartile values were calculated for minimum and maximum weekly physical activity. Frequency tables were used to determine the percentage of individuals at risk, and also for the stages of change data.

Analyses of co-variance (ANCOVA) were used to compare risk profiles within the various sectors, after co-varying for age and level of participation (%). Bonferroni post hoc analyses were used to determine which sectors were significantly different from each other. The relationship between demographic and clinical data and self-reported health status was investigated using Spearman's correlations. Chi-square analysis was used for the categorical nonparametric data. Chronological age and attendance were entered as covariates in all statistical analyses.

## Results

### Participant Characteristics

All employees were invited to attend the wellness days and the mean participation for the total sample was 25.82%, (women n = 812, men n = 623, total n = 1954) (Table 1). Participation varied according to sectors, and ranged from as low as 4% in the transport sector to 81% in the consulting sector (Table 1). Mean chronological age was also significantly different between sectors, with the manufacturing sector having the oldest participating employees. Both participation by the specific company and chronological age were entered as covariates in subsequent statistical analyses.

**Table 1:** Demographic characteristics of participants

	**Total (n = 1954)**	**Engineering (n = 223)**	**Finance (n = 851)**	**Consulting (n = 133)**	**Transport (n = 138)**	**Logistics (n = 42)**	**ITS (n = 173)**	**Manufacturing (n = 240)**	**Academic (n = 154)**
**% participants**	25.8	18.9	19.3	81.5	4.4	28.0	28.6	41.6	14.7
**% Women**	42	32	44	49	39	34	35	32	64
**Chronological Age (yrs)**	34.9 (± 10.3)	39.5 (± 10.70)	32.2 (± 9.45)	33.1 (± 8.3)	32.5 (± 9.3)	35.7 (± 8.6)	34.5 (± 10.4)	40.7 (± 10.58)	37.5 (± 10.0)
**Risk related Age (yrs)**	38.5 (± 12.9)	44.8 (± 14.1)	34.6 (± 11.3)	36.6 (± 10.60)	34.7 (± 10.0)	41.7 (± 12.7)	38.7 (± 12.3)	47.0 (± 13.6)	41.7 (± 12.7)

Results for chronological age and risk related age are reported as (mean ± SD)

Participation and women participating are represented as percentage.

### Clinical measures

#### Body Mass Index (BMI)

The mean BMI for the total sample was 25.5 ± 5.3, with 32% of the employees classified as overweight (25–29.9), 16% as obese (BMI ≥ 30) and only 44% considered normal weight (BMI ≥ 18.5 ≤ 24.9). Comparisons between sectors showed that the only sectors with a mean BMI which was normal weight were Consulting, Transport and ITS. After adjusting for age, the Logistic sectors had a significantly higher mean BMI compared to the Transport, Finance, ITS and Academic sectors (Table 2).

**Table 2:** Health and Lifestyle Characteristics of employees per business sector

	**Total (n = 1954)**	**Engineering (n = 223)**	**Finance (n = 851)**	**Consulting (n = 133)**	**Transport (n = 138)**	**Logistics (n = 42)**	**ITS (n = 173)**	**Manufacturing (n = 240)**	**Academic (n = 154)**
**BMI (kg/h^2^)**	25.5 (± 5.3)	26.9 (± 5.3)	25.7 (± 5.5)	24.8 (± 3.9)	24.7 (± 4.6)	28.8 (± 5.1)	24.9 (± 4.5)	26.4 (± 10.6)	25.9 (± 4.8)
**Cholesterol (mmol/l)**	4.5 (± 1.1)	4.5 (± 1.3)	4.6 (± 1.0)	4.3 (± 1.0)	4.36 (± 1.3)	4.7 (± 1.0)	4.4 (± 0.9)	4.6 (± 5.1)	4.3 (± 1.1)
**Systolic BP (mm Hg)**	121.9 (± 16.8)	129.4 (± 18.1)	125.1 (± 18.5)	121.0 (± 13.9)	122.3 (± 15.4)	120.4 (± 12.8)	127.4 (± 17.3)	137.6 (± 18.1)	122.2 (± 14.5)
**Diastolic BP (mm Hg)**	78.4 (± 11.2)	81.4 (± 10.4)	81.9 (± 13.5)	75.3 (± 12.0)	80.0 (± 10.2)	79.2 (± 13.3)	82.0 (± 10.1)	85.6 (± 12.1)	77.5 (± 9.6)
**Fruit and Vege (servings/day)**	2.7 (± 1.7)	2.7 (± 1.5)	2.56 (± 1.7)	2.9 (± 1.7)	3.1 (± 1.8)	2.5 (± 1.2)	2.7 (± 1.7)	2.4 (± 1.6)	2.7 (± 1.6)
**Average PA (min/wk)**	115.73 (± 131.0)	106.28 (± 129.14)	121.15 (± 136.36)	127.56 (± 142.35)	109.24 (± 128.4)	156.61 (± 184.43)	121.04 (± 119.64)	106.56 (± 121.51)	92.29 (± 105.67)

BMI: Body Mass Index; BP: blood pressure; vege: vegetables; Min PA: average minutes physical activity per week (self reported);

Results are reported as mean (± SD).

#### Cholesterol Concentrations

Nearly one-third (30%) of all participants chose not to have their cholesterol measured at the wellness day. Of those tested, the mean cholesterol concentration was 4.5 ± 1.13 mmol/l, reflecting an average low risk. The Finance sector employees had significantly higher cholesterol concentrations than either the Consulting and Academic sectors (Table 1).

#### Blood Pressure

Similar to the cholesterol results, the mean systolic and diastolic blood pressure results for the total sample (121.9 ± 5.3 mmHg and 78.4 ± 11.2, respectively) were normo-tensive. Although few (12%) of the participants were classified as hypertensive, 27% of the employees did not know their blood pressure status and chose not to have it measured at the wellness day.

### Lifestyle behaviour

#### Habitual fruit and vegetable intake

The majority of participants (86%) consumed less than the recommended five or more servings of fruit and vegetables per day, and mean daily intake was only 2.7 ± 1.7 serves, which was significantly different between sectors (p < 0.0001). The Transport sector recorded the highest number of daily servings, 3.1 ± 1.8 (Table 2), when compared to the other sectors.

#### Habitual Physical Activity

Average, minimum and maximum time spent weekly in moderate-to-vigorous physical activity was calculated based on self reported frequency, duration and intensity. The average time spent being physically active was 115.73 ± 131 minutes while the median minimum and maximum physical activity was 45 and 90 minutes per week, respectively. After adjusting for age and participation per company, the most physically active sector was Logistics while those employed in the Academic sector were the least physically active (p < 0.001).

### Age: Chronological and Risk-related age

**Figure 1:**
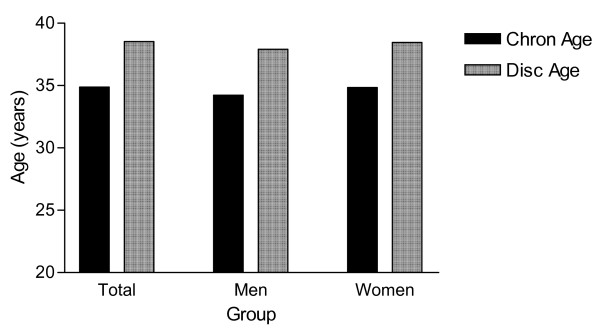
Chronological and Discovery Age for all employees (total), men and women.

The mean chronological age of the total sample of employees (n = 1951) was 34.9 ± 10.3 years. Risk-related age, calculated using the algorithm based on relative risks for lifestyle behaviour was significantly higher than chronological age for the total sample and for men and women when analysed separately (Figure 1). However, both chronological and risk-related age were significantly different between the sectors (P < 0.001), with the manufacturing sector having the oldest and finance having the youngest employees. Furthermore, risk-related age was negatively and significantly associated with the number of days away from work (r = -0.16; p < 0.001).

#### Risk-age difference

The Risk-age difference was calculated by subtracting the risk-related age from the chronological age. The risk-age difference was similar for men and women but significantly different between the business sectors. The difference between risk-related age and chronological age was greatest (p < 0.05) amongst the manufacturing sector (-6.86 years; p < 0.001) than the other sectors. Conversely, employees in the transport industry had significantly lower risk-age differences than the all the other sectors with the exception of consulting and finance.

The risk age difference was weakly, but significantly associated health-related costs associated with hospitalisation (r = -0.10; p = 0.02) and the use of chronic medication (r = -0.20; p < 0.001).

### Self-reported health status

Table 3 represents self-report data on the number of days that mental and physical health affected daily activities as well as the number of days absent from work for the previous month. Mental health affected daily performance more frequently than physical health, 4.0 ± 6.1 days and 2.3 ± 3.7 days, respectively. The mean number of days absent per month for the total sample was 2.2 ± 4.6 days.

**Table 3:** Self reported health status per sector

	**Total (n = 1954)**	**Engineering (n = 223)**	**Finance (n = 851)**	**Consulting (n = 133)**	**Transport (n = 138)**	**Logistics (n = 42)**	**ITS (n = 173)**	**Manufacturing (n = 240)**	**Academic (n = 154)**
**Phys Health **	2.3 ± 3.7	2.5 ± 4.7	2.4 ± 3.8	1.4 ± 2.4	1.5 ± 2.9	2.1± 3.1	1.4 ± 2.6	1.2 ± 2.7	2.4 ± 3.6
**Mental Health**	4.0 ± 6.1	3.3 ± 5.1	3.4 ± 5.8	3.5 ± 4.9	2.8 ± 5.6	4.9 ± 8.8	3.1 ± 5.4	2.9 ± 5.7	5.1 ± 7.8
**Days away work**	2.2 ± 4.6	1.9 ± 4.4	2.6 ± 5.0	1.3 ± 3.6	1.5 ± 3.9	2.5 ± 6.2	1.0 ± 2.3	0.9 ± 2.8	2.0 ± 4.5

Phys Health: number of days in the previous health where physical health adversely affected daily activities and performance; Mental Health: number of days in the previous health where physical health adversely affected daily activities and performance; Day away work the number of days absent from work due to ill health during the previous month.

There were significant inter-sectoral differences for self reported mental and physical health. Employees in the Engineering sector reported the highest number of days adversely affected by physical health (2.5 ± 4.7 days) compared to the Manufacturing sector who reported only 1.2 ± 2.7 days per month (Table 3).

The Academic sector reported the highest number of days per month adversely affected by mental health followed by the Logistics and Engineering sectors. Although the numbers of days adversely affected by physical and mental health were among the highest for the academic sector, these employees did not report the highest number of days absent from work. Employees in the Finance and Logistics sector reported a significantly greater number of work days lost during the preceding month than the other sectors, compared to the other sectors (Table 3).

### Readiness to change

One fifth of the employees who were consuming less than 5 servings of fruit and vegetables per day, in fact, believed that their habitual diet was already healthy and 37% reported that their diet was healthy with only occasional periods of unhealthy eating. Just under one-third (30%) of adults who did not consume sufficient fruit and vegetables expressed a desire to improve their nutritional habits.

On the other hand, nearly two thirds (62%) of those employees who were identified as insufficiently active expressed a desire to increase their weekly levels of habitual physical activity, 11% reported that they were doing sufficient exercise, while 23% indicated that they were not intending to become more active. A greater proportion of men wanted to increase their habitual levels of physical activity compared to women (70% versus 57%).

Most of the employees classified as overweight (BMI > 25) indicated that they would like to lose weight. Nearly half (40%) reported they wanted to lose 10 kg, 28% wanted to lose 5 kg and 13% wanted to lose 2 kg. However 15% of the overweight employees reported that they felt they were an ideal weight, or that they wanted to gain weight. Significantly more women (85%) than men (73%) reported that they intended to lose between 2 and 10 kg.

Of those employees who smoked, 35% did not intend quitting, while 56% intended to stop smoking in the following 12 months. Less than half (45%) of the women wanted to quit smoking compared to 65% of the men.

### Relationship between risk factors

The percentage of participants per business sector classified as 'at risk' for smoking, fruit and vegetable intake, BMI, habitual physical activity and serum cholesterol concentration is shown in Table 4. The most prevalent risk factors were low levels of average weekly physical activity and daily fruit and vegetable intake. More than 60% of men and women were not meeting public health recommendations for health-enhancing physical activity (>150 min/week). Similarly fewer than 20% were meeting the daily dietary recommendations for fruit and vegetable intake (<5 units per day).

**Table 4:** Percentage of participants classified as 'at risk' for each of the business sectors [34]

	**BMI (≥ 25)**	**Cholesterol (>5 mmol/l)**	**Systolic BP (≥ 140 mm Hg)**	**Diastolic BP (≥ 90 mm Hg)**	**Fruit and Veg (< 5/day)**	**Max PA (< 150 min/wk)**	**Smoking (current smoker)**
**Total sample (n = 1954)**	48.6 (950)	18.6 (363)	13.0 (254)	12.2 (238)	86.3 (1687)	69 (1351)	19.9 (389)
**Eng (n = 223)**	58.7 (131)	23.8 (53)	18.8 (42)	16.6 (37)	88.3 (197)	73 (162)	14.8 (33)
**Fin (n = 851)**	44.5 (379)	10.6 (90)	8.2 (70)	7.9 (67)	86.0 (732)	69 (583)	14.2 (121)
**Cons (n = 133)**	45.1 (60)	21.1 (28)	3.7 (5)	5.3 (7)	82.0 (109)	62 (82)	27.1 (36)
**Trans (n = 138)**	44.9 (62)	25.4(35)	10.1 (14)	14.5 (20)	79.0 (109)	72 (100)	18.8 (26)
**Log (n = 42)**	76.1 (32)	28.6 (12)	4.8 (2)	7.1 (3)	95.2 (40)	62 (26)	33.3 (14)
**ITS (n = 173)**	39.9 (69)	19.7 (34)	15.0 (26)	17.3 (30)	88.4 (153)	61 (106)	28.9 (50)
**Man (n = 240)**	56.7 (136)	32.5 (78)	35.8 (86)	26.3 (63)	90 (216)	71 (170)	29.6 (71)
**Aca (n = 154)**	52.6 (81)	21.4 (33)	5.8 (9)	7.1 (11)	85.1 (131)		24.7 (38)

BMI: Body Mass Index; BP: blood pressure; vege: vegetables; Max PA: maximum number of minutes per week of physical activity (self reported)

**Figure 2:**
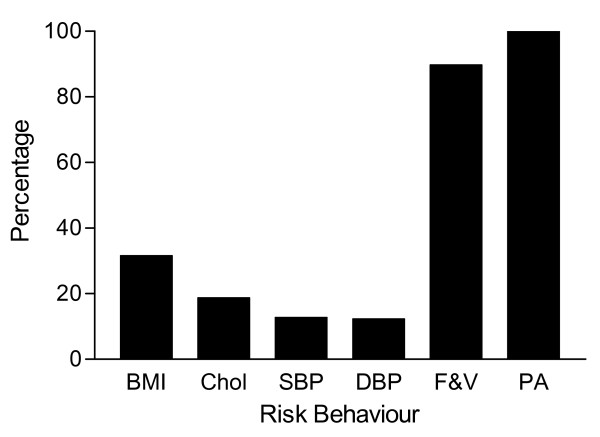
Clustering of risk factors: Percentage of Physically inactive and additive risk factors for total sample.

One third of the participants (33%) had at least 2 risk factors with 20% having 4 or more risk factors. When examining the clustering of additional risk factors with insufficient physical activity, a similar pattern was observed (Figure 2). Of those employees who were at risk for NCD due to insufficient physical activity, 47% and 22% had additional two and three risk factors, respectively. Participants who were not physically active, nearly a third (31.5%) of the employees were also overweight or obese, 20% were smokers, 19% had blood cholesterol concentrations more than 5 mmol/l and 12% were hypertensive. Furthermore, 90% of those who were inactive were also consuming inadequate fruit and vegetable servings per day.

## Discussion

This study aimed to evaluate an existing wellness programme and to assess the distribution of lifestyle and clinical risk factors in an employed adult population, voluntarily presenting for health risk appraisal. The first important finding was the low percentage of participation in a health risk appraisal offered on site at work among employees (mean 26%) and that participation varied significantly different between sectors. Despite the low level of participation, our rates were similar to that reported in a comprehensive workplace intervention which gave employees the opportunity to participate in a variety of health promotion programmes [8]. The study by Aldana et al was conducted over 2-years with 23% of the employees participating in the first year and 20% during the second [20]. Similar to our findings, the majority of their participants were female.

A posteriori analyses was conducted in order to determine if the results differed between companies with higher response rates, compared to those with lower response rates. We divided the companies into tertiles based on participation, and then conducted an analyses of variance (ANOVA) to determine if there were significant differences in the health and behaviour profile according to levels participation. There were no significant differences in age, both chronological age and risk-related age, Body Mass Index (BMI), cholesterol concentration, and habitual levels of physical activity in the high versus the low responders. Conversely, the companies with the highest response rates had significantly higher blood pressure, more total risk factors for NCD and lower daily fruit and vegetable consumption than those with the lowest response rates. Thus the wellness days in the companies with the highest attendance seemed to be attracting employees at increased risk of non-communicable diseases. It is therefore important to address attendance and uptake in worksite-based programmes. This is supported by Kwak et al who advocates the reporting of participation rates in order to correctly interpret results and implications of the research findings [9].

It has been previously demonstrated that the success of recruiting employees in health risk screening is influenced by the interest shown from the company's management, as well as, internal advertising and marketing strategies [21,22]. In these 18 companies, low levels of participation may be a reflection of poor marketing and advertising prior to the wellness days. The poor uptake could also be attributed to availability of staff on the wellness day, which was only offered approximately once per year. For example, the transport sector comprised of airline companies, and the low level of participation may be in part due to the fact that some staff was not available on the wellness day. The solution in this case, would be to offer the health and wellness events on more than one day per year in order to attract more participants.

Since one of the aims of these wellness days is to offer individuals an opportunity to determine their health status, particularly those at increased risk, the advertising plays an important role in attracting as many volunteer participants as possible. Leslie et al. used e-mail communication to increase participation in workplace-based health intervention programmes [23], which was followed up with a telephone call resulting in an uptake of 79% [23]. Other factors which could increase participation is the provision of incentives [24] and also the establishment of employee advisory boards who have management involvement, a level of autonomy and also company commitment [25]. Therefore, the recruitment for wellness days, particularly if they are only one-day events, should begin earlier than the one-week time period used in the current study and should also consider including direct contact with the employee.

Participation in workplace based interventions which include health risk assessments, has also been associated with decreased short term disability days away from work [26]. Serxner et al found that employees who participated in a health risk assessment at least once per year significantly decreased days away from work by 5%, compared to a 15% increase among non-participants [26]. The potential benefits of increasing participation would therefore include future costs associated with absenteeism and training temporary personnel. Bias in interpretation was subsequently addressed by adjusting inter-sectoral comparisons for percentage participation in the health risk appraisal.

The second important finding in our study was that risk-related age was significantly higher than chronological age and this risk-age difference was greatest in the Manufacturing sector. The risk-related age is a reflection of the presence of risk factors such as smoking, inadequate physical activity and fruit and vegetable intake, elevated cholesterol and a BMI greater than 24.9 kg/m^2^. These are all modifiable risk factors, and comprehensive interventions may reduce risk-related age and possibly result in a 'gain' in years [27,28,8]. The inter-sectoral differences may be used by health insurers in identifying companies which are likely to benefit most from intervention programmes. Improved health status and lifestyle habits has been associated with reduction in health care costs, increased productivity and decreased absenteeism [29,28,8]. These findings are supported by Serxner et al. 2001 who found that employees who have high blood pressure, high cholesterol and are overweight have higher rates of absenteeism and also related medical costs [27].

This research study is among the few in South Africa (SA) which allows for the comparison of the health status of the corporate sector to that of the general SA population. This is due in part, to the fact that the South African Demographic Health survey was recently completed in 2002–2003. Secondly, measures were, in some instances, comparable, due to the similarity of definition of risk factors and risk questionnaires between the two surveys, as well as risk cut-points. For example, the men in the present study had a higher prevalence of overweight and obesity compared to the general male population [2]. Conversely, women in the present study had a lower prevalence of overweight and obesity, compared to that of the SADHS. The differences between the employed adults and general population may be due to differences in educational and socio-economic status. The SADHS reported that the prevalence of overweight was highest among men in urban settings and those who were educated [2], which reflects the participants in the current study. In addition, SAHDS showed that women with the lowest levels of education were the most obese [2], while most of the women in our study had some form of higher education.

Furthermore, the individuals screened as part of this study report lower levels of participation in physical activity than the general population. This has important implications since physical activity is associated with decreased risk of disease, but also, with greater levels of productivity and lower rates of absenteeism, which is important in the corporate sector [14]. Indeed, our results showed that those with higher levels of weekly physical activity reported significantly fewer days away from work the previous month. This is supported by Jacobson and Aldana (2001) who investigated the relationship between the frequency of aerobic activity and illness-related absenteeism among US workers (n = 79 070) representing 250 worksites [14]. Even only one day of physical activity per week was associated with significantly reduced absenteeism compared to those employees who were inactive, and further reductions were observed when comparing 2 days to one day of physical activity [14].

This research study has also shown a tendency for the clustering of risk factors and a large percentage of our participants had 4 or more risk factors (88%). Data from the 'StayWell' programme showed that employees with 4 or more risk factors were 1.75 times more likely to have higher absenteeism rates than those with fewer risk factors [27]. Our study corroborates these findings where those with a higher number of risk factors had significantly more days away from work, and also more days with performance adversely affected by poor mental or physical health. Therefore, it is likely that reducing the number of risk factors will have important implications in worksite settings, reducing both the direct and indirect costs associated with absenteeism.

Clustering of risk factors has also been associated with diseases such as hypertension, heart disease and diabetes [30]. Results from our study shows that insufficient physical activity was coupled with the presence of additional risk factors among employees such as insufficient fruit and vegetable intake (80%), smoking (61%), overweight or obese (31%), increased serum cholesterol concentration (19%) and elevated blood pressure (12%). These results suggest that increasing habitual physical activity may positively impact on the other risk factors, and subsequently lower the overall risk profile of individuals. Indeed, previous research has suggested that physical activity may act as a catalyst and entry point for improving diet and stopping smoking [10]. Reductions in risk factors that could therefore potentially be achieved by increasing habitual physical activity could decrease the risk of morbidity and mortality [26]. In addition, by decreasing the total number of risk factors, the total number of days absent from work can also be decreased [27].

The health risk assessment has been regarded as an entry point in comprehensive health promotion programmes and precedes the implementation of targeted interventions [31]. Completing a self-reported questionnaire such as the one used in our study to determine the prevalence of risk factors and to calculate risk-related age, may increase an individual's awareness of risk factors and aspects of their life that could improve. This increased awareness could be the first step in initiating change and improving health status, and risk-related age. Pelletier et al (2004) administered an online health risk appraisal on behalf of a health care provider to employees at baseline and again one-year later [32]. No other interventions were reported in their study, yet there were significant risk reductions observed for dietary habits, elevated serum cholesterol, and non-significant reductions in inactivity and BMI [32]. The potential health and cost benefits that can be obtained by following up the health risk assessment with an intervention could be greater than by only offering screening activities. Thus, interventions could be targeted successfully at those categorised as 'high risk' or 'moderate risk' [31] since those in the higher risk categories could show greater improvements in health [12].

Another important finding in our study was there was a knowledge-behaviour "gap", with a large percentage of employees believing that their dietary habits were healthy, despite consuming less than the recommended 5 servings of fruit and vegetables per day. These results highlight the gap in knowledge and/or awareness and the need for interventions which include an educational component. This is supported by Cook et al's (2001) findings where a higher nutrition knowledge score was associated with increased vegetable intake, and belief in healthy nutrition was reflected with increased fruit consumption [33].

Health risk has been associated with both increased presenteeism and absenteeism [32]. Thus, another noteworthy finding from our study was that the total number of days adversely affected by poor mental or physical health was less than the number of days away from work. These results may provide an indirect indication of presenteeism, suggesting that the employee is at work, but experiencing low levels of productivity. It has been widely established that health and well-being impact on work performance and job satisfaction [34].

Another important outcome of this study was that it allowed for inter-sectoral comparisons for risk factors and self reported health status. There were significant differences for each of the health and lifestyle measures among the various sectors. However, no single sector consistently emerged as having the healthiest or least healthy employees. Consequently, intervention strategies should be based on the individual requirement or health status of the various sectors or companies.

### Limitations

The main limitation of this study is that employees volunteer to participate in the wellness days, thus our results may be biased towards those willing to participate. However, Goetzel et al. reported that there were no significant differences in the average number of risks at baseline between participants and non-participants in Johnson and Johnson's Health and Wellness programme [12].

We acknowledge that this was an opportunistic study, evaluating an existing wellness programme. However the findings do provide some insight into the health profile of the South African corporate population, and underscores the need for further, and more representative research.

## Conclusion

Despite a decreased risk for non-communicable disease based on chronological age among the participants in our study, their risk is increased based on the presence of other risk factors such as inactivity and increased BMI. Furthermore, based on the higher rates of overweight and obesity, smoking and inactivity; men presenting for worksite wellness days may actually be less healthy than the general SA public. The relationship for women is slightly different, since they have a lower rate of overweight and obesity and smoking than that shown in the SADHS, but they have much higher levels of inactivity.

This has important health and economic consequences, and underscores the importance of implementing strategies and interventions aimed at improving the health status of employees. Intervention programmes targeting these risk factors would therefore play an important role in improving the health profile of employees.

## Competing interests

The UCT/MRC Exercise Science and Sports Medicine Research Unit (ESSM) receives research funding from the national health insurer, Discovery Health. Discovery Health funded the data collection component of the study, especially since it forms part of their corporate offering. The data analysis and preparation was covered by an annual grant from Discovery Health to ESSM.

However, in terms of the agreement between the funder and ESSM, ESSM are free to publish all research findings.

## Authors' contributions

TLK–A was involved in the conception and design of research study, data cleaning and analysis, and also in drafting and writing manuscript. CB assisted with data collection, and also the cleaning and analysis of some of the data, and in the writing and editing of the manuscript. CN worked for the health insurance company and played a role in collecting and collating the health risk assessment data and acquisition of health related claims, and also edited the manuscript. LD worked for the health insurance company and played a role in collecting and collating the health risk assessment data and acquisition of health related claims, and also edited the manuscript. TDN played role in critically revising and editing the manuscript. EVL was involved in the conception and design of research study, assisted and guided the statistical analysis, in the writing and editing of the manuscript and also the general management of the research team.

## Pre-publication history

The pre-publication history for this paper can be accessed here:


